# Impact of different leaf velocities and dose rates on the number of monitor units and the dose-volume-histograms using intensity modulated radiotherapy with sliding-window technique

**DOI:** 10.1186/1748-717X-3-31

**Published:** 2008-09-23

**Authors:** Hilke Vorwerk, Daniela Wagner, Clemens F Hess

**Affiliations:** 1 Department of Radiotherapy and Radiooncology, University Hospital Göttingen, Robert-Koch-Str. 40, 37099 Göttingen, Germany

## Abstract

**Background:**

Intensity modulated radiotherapy (IMRT) using sliding window technique utilises a leaf sequencing algorithm, which takes some control system limitations like dose rates (DR) and velocity of the leafs (LV) into account. The effect of altering these limitations on the number of monitor units and radiation dose to the organs at risk (OAR) were analysed.

**Methods:**

IMRT plans for different LVs from 1.0 cm/sec to 10.0 cm/sec and different DRs from 100 MU/min to 600 MU/min for two patients with prostate cancer and two patients with squamous cell cancer of the scalp (SCCscalp) were calculated using the same "optimal fluence map". For each field the number of monitor units, the dose volume histograms and the differences in the "actual fluence maps" of the fields were analysed.

**Results:**

With increase of the DR and decrease of the LV the number of monitor units increased and consequentially the radiation dose given to the OAR. In particular the serial OARs of patients with SCCscalp, which are located outside the end position of the leafs and inside the open field, received an additional dose of a higher DR and lower LV is used.

**Conclusion:**

For best protection of organs at risk, a low DR and high LV should be applied. But the consequence of a low DR is both a long treatment time and also that a LV of higher than 3.0 cm/sec is mechanically not applicable. Our recommendation for an optimisation of the discussed parameters is a leaf velocity of 2.5 cm/sec and a dose rate of 300–400 MU/min (prostate cancer) and 100–200 MU/min (SCCscalp) for best protection of organs at risk, short treatment time and number of monitor units.

## Background

Intensity modulated radiotherapy (IMRT) offers a method of delivering a radiation dose conformed to the shape of targets while minimizing the dose to the surrounding tissue and nearby critical organs. For IMRT with sliding window technique, modern IMRT planning systems incorporate many control system limitations in their leaf sequencing algorithms, such as limits for the leaf velocity (LV), the actual dose rate (DR), leakage, dynamic leaf gap, transmission and a minimum leaf gap (mechanical distance between the tops of the MLCs). Incorporation of control system limitations into the leaf sequencing algorithm results in decreased discrepancies between planned and delivered IMRT fields. Dynamic sliding-window leaf sequences can be produced, which require neither beam interruptions nor dose rate modulations for the parameter values used in calculating the sequence [1,2].

In the treatment planning process for IMRT with sliding window technique, an optimization process is first carried out to optimise the coverage of the target volume and to protect the organs at risk. With this optimization information the system calculates an "optimal fluence map". In a second step the leaf motions are calculated taking the above mentioned limitations of the real accelerator into consideration. Thus "actual fluence maps" are created out of the "optimal fluence maps". The used control system limitations can be defined by the user.

The purpose of our study was to analyse the impact of different DR and LV on the "actual fluence maps" using the same "optimal fluence map" and thereby the impact on the number of monitor units and the dose-volume-histogram of the organs at risk (OAR). We found larger impacts on the results for patients with squamous cell cancer of the scalp than for patients with prostate cancer.

## Methods

### Patient data

**Figure 1:**
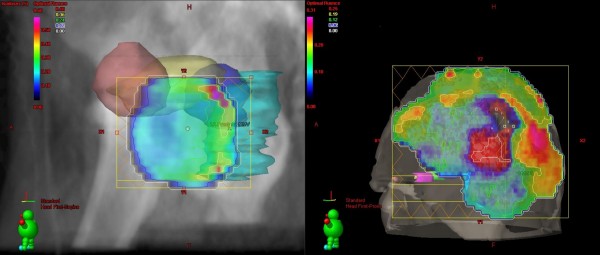
**Beam eye view illustrations of "optimal fluences" for patients with prostate cancer (left) and squamous cell cancer of the scalp (right)**. Left: Beam eye view from 305° gantry angle from a patient (PC) in relation to the locations of the organs at risk: femoral heads (red and blue), rectum (cyan), bladder (yellow). Right: Beam eye view from 290° gantry angle from a patient (SCCscalp) in relation to the locations of the organs at risk: chiasm (blue), brainstem (cyan), nn. optici (green and purple), lenses (yellow and orange).

Patient data was acquired from four patients who were recently treated with IMRT in our department. Two of the patients were treated for prostate cancer (PC) with 72 Gy (2 Gy per fraction) using five fields and two for squamous cell cancer of the scalp (SCCscalp) with 60 Gy (2 Gy) per fraction using four to five fields (figure 1). We chose two examples per tumor entity to avoid random errors in one patient.

### Treatment planning system

For this study the planning system Helios (Version 8.1 ARIA, Algorithms 8.1.17, Varian Medical Systems, Palo Alto, CA, USA) was used. The treatment plans were calculated for a Clinac 2300 C/D from Varian Medical Systems and for a Millenium multi leaf collimator (MLC) with 120 leafs.

The treatment planning system created "optimal fluence maps" after the optimization process (figure 1). These fluences did not consider the mechanical components of the linear accelerator and MLC. Particularly the "optimal fluences maps" were independent of the LV and the DR. In a second step the leaf motions were calculated based on the chosen LV and DR of the field (see below). Taking the leaf motions into consideration, the treatment planning system created "actual fluence maps", which contain control system limitations of the accelerator in contrast to the "optimal fluence maps". The dose distribution was calculated in a third step based on the "actual fluence".

### Analysis

We analysed the number of monitor units for each field depending on following DR and LV:

• 1.0 cm/sec, 1.5 cm/sec, 2.0 cm/sec, 2.5 cm/sec, 3.0 cm/sec, 3.5 cm/sec, 4.0 cm/sec, 10.0 cm/sec

• 100 MU/min, 200 MU/min, 300 MU/min, 400 MU/min, 500 MU/min, 600 MU/min

**Figure 2:**
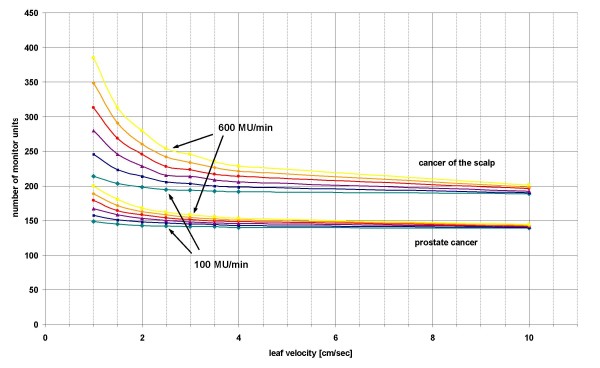
**Influence of different maximum leaf velocities on the number of monitor units applied**. Mean of the number of monitor units of all fields against the leaf velocity of both patient with prostate cancer and of both patient with squamous cell cancer of the scalp for different dose rates: green rhombus – 100 MU/min, blue square – 200 MU/min, purple triangle – 300 MU/min, red circle – 400 MU/min, orange rhombus – 500 MU/min, yellow square – 600 MU/min.

IMRT plans with a leaf velocity limit of 10.0 cm/sec are mechanically not applicable. We chose this velocity in order to analyse the theoretical effects of very high LV (figure 2).

We measured the number of monitor units (NMU) depending on the DR and LV for all fields and entities. The figures demonstrate examples.

### Different tumor entities and different photon energies

For patients with SCC scalp we used a four and five field technique with 6 MVX (photons with a maximum energy of 6 MeV). The patients with PC were treated with a mixed beam technique, which acquired two fields with 20 MVX from the lateral sides and three fields from dorsal and ventral with 6 MVX. All fields with 6 MVX were analysed and in a second step the fields with 6 MVX were compared with the 20 MVX fields.

### Effects on the dose volume histogram

The influence of the different LV and DR on the dose volume histograms (DVH) of the planning target volume (PTV), the body and OAR were analysed for all fields and "actual fluence maps". The bladder, rectum, and femoral heads were considered as OAR for the patients with PC (figure 1). All of these OARs were located in the direct course of beam of at least two of the five fields.

The chiasm, brain stem, nn. optici, lenses, and brain were analysed for the patients with SCCscalp (figure 1) [3]. In these patients the chiasm, brain stem and nn. optici were located in the direct course of beam of at least two fields. The lenses are always located outside the end positions of the leafs and in half of the fields inside the open field.

### Comparison of actual fluence images

The treatment planning system has no function included to compare different fluence images, but the planning system can create artificial predicted portal dose images (PPDI). This tool can be used for the verification of IMRT plans. PPDIs of the "actual fluence maps" were created and compared with each other by use of the gamma index evaluation with 3 mm and 3% [4-6]. The relation between the gamma index and the number of monitor units were analysed.

## Results

### The number of monitor units depends on the dose rate

**Figure 3:**
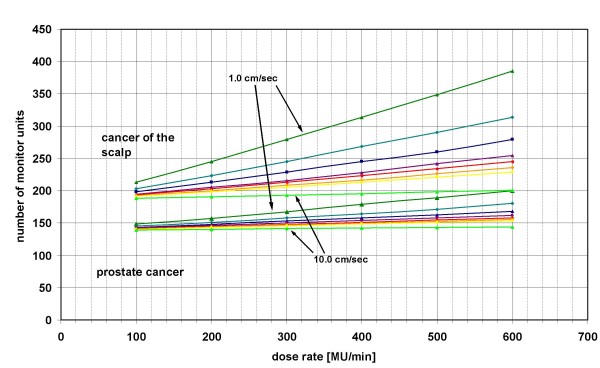
**Influence of different dose rates on the number of monitor units applied**. Mean of the number of monitor units of all fields against the dose rate of both patient with prostate cancer and of both patient with squamous cell cancer of the scalp for different leaf velocities: dark green triangle- 1.0 cm/sec, turquoise rhombus – 1.5 cm/sec, blue square – 2.0 cm/sec, purple triangle – 2.5 cm/sec, red square – 3.0 cm/sec, orange rhombus – 3.5 cm/sec, yellow circle – 4.0 cm/sec, light green triangle – 10.0 cm/sec.

The higher the DR the higher was the NMU per field (NMU ~ DR) not only for the two treatment plans of patients with PC but also for the two treatment plans of patients with SCCscalp (figure 3). With increase of the LV the gradient of the straight line became steeper for all fields, particularly for patients with SCCscalp (figure 3).

### The number of monitor units depends on the leaf velocity

The higher the DR, the higher the difference between the NMU for different LV (figure 3). The NMU decreased with higher LV approximately as followed (figure 2):

$$\begin{array}{lll} NMU=c_{1}LV^{c_{2}} & {\text{c}}_{\text{1}}=140\text{ to }193 & {\text{c}}_{2}=-1.6\text{ to -}0.3\text{ for different DR }(\text{PC})\\ & {\text{c}}_{1}=212\text{ to }373 & {\text{c}}_{2}=-0.3\text{ to -}0.05\text{ for different DR }(\text{SCCscalp}) \end{array}$$

With a leaf velocity of 10.0 cm/sec the differences between the NMU for different DR was very low with 3–7 MU for PC and 6–14 MU for SCCscalp.

The dependence of the NMU on the LV was more pronounced in treatment plans for patients with SCCscalp than with PC (figure 2 and 3). The mean of the NMU of all fields were higher for SCC scalp (225 MU) than for PC (199 MU).

To estimate the complexity of the fluence maps for all fields, the NMU of the dynamic field was divided by the NMU of the corresponding open field. The complexity of the fluence maps was higher in the fields of SCCscalp compared with the fields of PC as expected.

### Comparison of fields with 6 MVX and 20 MVX in treatment plans for prostate cancer

**Figure 4:**
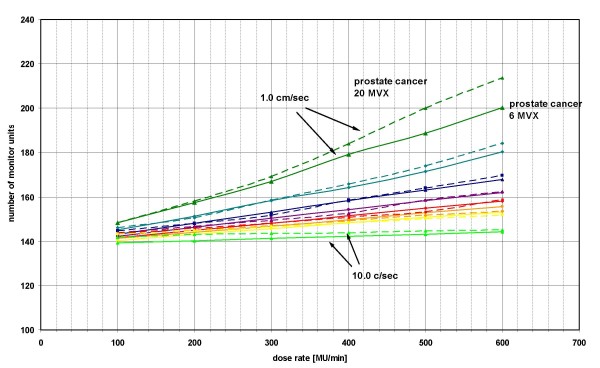
**Influence of different energies on the number of monitor units applied**. Mean of the number of monitor units of all fields against the dose rate of all fields with 6 MVX and all fields with 20 MVX of both patients with PC for different leaf velocities. The continuous lines are values from the field with 6 MVX and the dashed lines from the field with 20 MVX. Calculation was done for different leaf velocities: dark green triangle- 1.0 cm/sec, turquoise rhombus – 1.5 cm/sec, blue square – 2.0 cm/sec, purple triangle – 2.5 cm/sec, red square – 3.0 cm/sec, orange rhombus – 3.5 cm/sec, yellow circle – 4.0 cm/sec, light green triangle – 10.0 cm/sec.

The gradient of the linear dependence between NMU and DR was comparable for fields planned with 6 MVX and 20 MVX (figure 4). Only for a LV of 1.0 cm/sec and 20 MVX could we demonstrate a higher NMU than expected (median 75 MU's difference between all fluences of all fields with 6 MVX (PC) and of all fields with 20 MVX (PC)). Unlike all other curves there was nearly no dependence of the NMU on the DR for a leaf velocity of 10.0 cm/sec and 20 MVX.

### Influence of different leaf velocities on the dose-volume-histogram in prostate cancer

**Figure 5:**
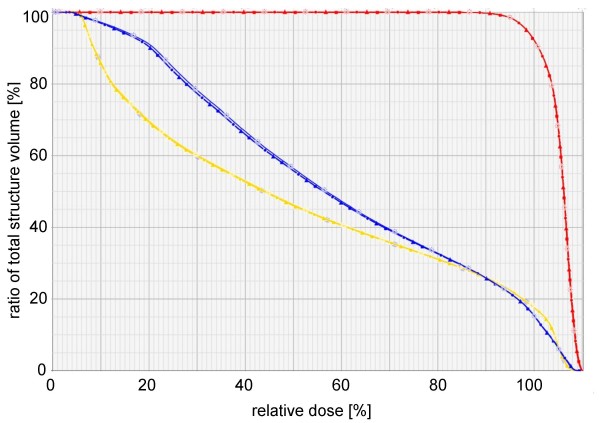
**Dose volume histogram of a patient with prostate cancer**. Dose volume histogram (relation of relative volume to delivered relative dose) from one patient for PTV (red), rectum (blue) and bladder (yellow) for different dose rates (DR) and leaf velocities (LV): circle – DR 100 MU/min and LV 1.0 cm/sec. triangle – DR 100 MU/min and LV 10.0 cm/sec. sun – DR 600 MU/min and LV 1.0 cm/sec. square – DR 600 MU/min and LV 10.0 cm/sec.

Different LV and DR had little influence on the coverage of the PTV (up to 0.6% difference relative to the mean over all DR and LV), the dose exposition of the OAR (figure 5) and the body (represents the low dose areas). The higher the DR and the lower the LV, the higher was the dose given to the PTV and OAR. The D55 and D90 of the rectum differed by 0.2% – 3.7% and 0.3% – 3.9% relative to the mean over all DR and LV, respectively. This was comparable for the bladder, the femoral heads and the body.

### Impact of different leaf velocities on the dose-volume-histogram in patients with squamous cell cancer of the scalp

The PTV coverage for both patients was nearly independent of the DR and LV. The percentage of the volume covered by the 100% isodose line deviated up to 0.25% from the mean over all DR and LV for each of both patients.

**Figure 6:**
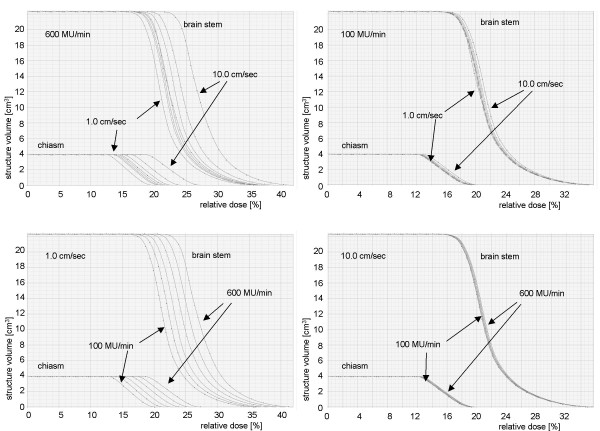
**Impact of different maximum leaf speeds and dose rates on the dose volume histograms**. Dose volume histogram (relation of absolute volume to delivered dose) for the chiasm and brain stem for different dose rates (triangle – 100 MU/min, square – 200 MU/min, sun – 300 MU/min, circle – 400 MU/min, rhombus – 500 MU/min, heart – 600 MU/min) and leaf velocities (circle – 1.0 cm/sec, sun – 1.5 cm/sec, "B" – 2.0 cm/sec, "A" – 2.5 cm/sec, square – 3.0 cm/sec, rhombus – 3.5 cm/sec, heart – 4.0 cm/sec, triangle – 10.0 cm/min) for one patient with squamous cell cancer of the scalp. Top left – dose rate of 600 MU/min and different leaf velocities. Top right – dose rate of 100 MU/min and different leaf velocities. Bottom left – leaf velocity of 1.0 cm/sec and different dose rates. Bottom right – leaf velocity of 10.0 cm/sec and different dose rates.

The radiation dose given to all OAR in patients with SCCscalp showed an increase with increased NMU and DR and with decreased LV (figure 6). For the chiasm, the brain stem and the nn. optici the maximum dose deviated by 6.0% – 9.1% (= 3.1 Gy – 4.8 Gy) for different DR and LV relative to the mean of the maximum dose of all fluences (all DR and LV) of each patient. The dose never exceeded the dose limitations of the OAR (54 Gy), because the absolute given doses (maximum 25.2 Gy) were about half of the limits for the OAR.

The maximum dose to the left and right lens of the first patient with SCCscalp ranged between 2.0 Gy to 4.0 Gy and 4.4 Gy to 8.3 Gy, respectively. In this patient the critical dose of 6 Gy to the lenses was exceeded by use of a high DR and low LV (LV 1.5–2.0 cm/sec and DR 500–600 MU/min, LV 1.0 cm/sec and DR 300–600 MU/min). We detected comparable results for the second patient with SCCscalp.

### Gamma index of predicted portal dose images

The "actual fluence maps" differed for different DR and LV while the "optimal fluence map" was the same. The comparison between the "actual fluence maps" calculated using a DR of 100 MU/min and 600 MU/min showed a mean gamma index over all fields of 1.1 (PC) and 2.7 (SCCscalp) for a LV of 1.0 and of 0.2 (PC) and 0.7 (SCCscalp) for a LV of 10.0. The NMU was correlated with the gamma index – the higher the NMU, the higher the gamma index (figure 2).

The mean gamma index between the fields with a LV of 1.0 cm/sec and 10.0 cm/sec was PC (SCCscalp) 0.2 (0.8) and 1.1 (2.9) using a DR of 100 MU/min and 600 MU/min, respectively. The gamma index was correlated to the NMU as well (figure 3).

The images of the gamma evaluation showed differences particularly outside of the end positions of the leafs but inside of the open fields.

## Discussion

According to our analysis the NMU in IMRT plans with sliding window technique increased with decreasing LV (NMU ~ LV^-1^) and increasing DR (NMU ~ DR) (figure 2 and 3). The NMU are important for the amount of transmitted radiation dose through the MLC and the treatment time (time ~ NMU).

The treatment time should be short in order to reduce the effects induced by patient movement, breathing and other random errors during treatment. A low DR implicates long treatment times, even though the NMU is lower than for a high DR. To decrease the treatment time the DR should be as high as possible.

For treatment planning of IMRT the reduction of the NMU is an important factor. The higher the NMU, the higher is the transmitted radiation dose [7]. This depends on the transmission passing through the leafs, between two adjacent leafs of one carriage and through the gap between two opposite leafs (this is more pronounced by the Varian MLCs compared to MLC of other companies because of their rounded leaf edges). Decreasing the transmitted radiation doses is important for protection of the organs at risk, especially for organs that are located outside the end position of the leafs and inside the open fields as the lenses (figure 1).

To minimise the NMU and to avoid unnecessary radiation doses given to the OAR, the DR should be as low as possible. Thus the choice of the DR must be a compromise between these considerations.

We compared the actual fluences of the IMRT fields created after leaf sequencing for different LV and DR, to detect differences in the fluence distribution and particularly the location of these differences. The maximum gamma index was higher for fluences with a higher NMU as an expression of transmitted and scattered radiation. Most differences could be detected outside the end position of the leafs and inside the open field. This correlated with a higher radiation dose in the OAR, which were located outside the end position of leafs and inside the open field, as in the lenses in patients with SCCscalp. This has a significant impact because normally the radiation dose decreases exponentially with increasing distance from the treatment field [8]. But in this case the OAR received more radiation dose than expected when looking at the distance to the endpoints of the MLC.

The coverage of the PTV is approximately independent of the LV. Paying attention to the optimal protection of the OAR the use of a high LV would be the best choice. On the other hand a high LV implicates mechanical problems during application of the radiation dose. This may result in unintended beam interruptions and dose rate modulations during application. A high leaf velocity results in more frequent collisions between the MLC with more malfunctions of the MLC motors as a consequence. An avoidance of collisions can be inducted by larger leaf gaps (minimum distance between the leave tops) [1]. Furthermore, with a large leaf gap the amount of transmitted radiation dose between the two MLC banks will increase. Because of these mechanical and technical limits the LV should not exceed 3.0 cm/sec.

As desired the higher the LV, the lower are the NMU, the treatment time and the given radiation dose to the OAR. In consequence we recommend a LV of 2.5 – 3.0 cm/sec.

The intension by the DR choice is to find the best compromise between reducing treatment time using a high DR and reducing the given dose to the OAR and the NMU using a low DR at the same time.

### Limits for patients with prostate cancer

In patients with PC the effects on the OAR are not noticeably higher with use of a high DR than with a DR of 100 MU/min, but the NMU increases. To decrease the NMU (with a low DR) and the treatment time (with a high DR) we recommend a DR of 300 – 400 MU/min.

By use of a LV of 2.5 – 3.0 cm/sec and a dose rate of 300 – 400 MU/min the implication of fields with 6 MVX or 20 MVX is negligible concerning the NMU (figure 4). In order to make the decision regarding the energy used, one should consider other reasons such as lowering the maximum dose or a reduction of the production of neutrons.

For high DR and low LV a high increase of the NMU for fields with 20 MVX was detected. For fields with 6 MVX and 20 MVX the limitations slip in the sequencing algorithm are the same except for the transmission and dynamic leaf gap. The higher NMU can only be founded in these parameters.

### Limits for patients with squamous cell cancer of the scalp

The NMU is higher for patients with SCCscalp than for patients with PC. This may be due to larger field size or higher complexity of the fields of patients with SCCscalp. Even if the equivalent square of the radiation fields were larger for SCCscalp than for PC, we assume that the complexity of the fluences had the most important influence on the NMU [9].

For the patients with SCCscalp the effects on the OAR are more dependent on the used DR than for patients with PC. This may result not only from the higher NMU but also from the location of the OAR. In our analysis we found maximal radiation doses to the lenses, which varied between 2.0 Gy and 8.3 Gy for different LV and DR. The TD 50/5 for lenses is 6 Gy [3], so that the use of different specifications for planning leads to acceptable or unacceptable dose given to the OAR. The OAR for patients with PC are located largely inside the radiation fields, so that the increased transmitted and scattered radiation dose does not effect the patients with SCCscalp as much, whereas the OAR were mostly located outside the end position of the leafs. In these cases the choice of the LV and the DR is more important than for PC, because the influence on the NMU and transmission radiation is very high. Another alternative to reduce transmitted radiation through the MLC may be the use of jaws, which follow the open window of the MLC dynamically to reduce the delivered dose outside the open field as described by Schmidhalter et al. [7].

To reduce radiation dose given to the OAR a lower DR than for patients with PC should be selected to decrease the NMU and therefore the amount of transmitted and scattered radiation dose. Therefore we recommend a DR of 100 – 200 MU/min.

## Conclusion

To decrease treatment time, NMU and radiation dose given to the OAR in IMRT planning with sliding window technique, a careful decision for DR and LV should be performed. Under consideration of the above mentioned and additionally mechanical and technical aspects limits for the LV of 2.5 – 3.0 cm/sec and for the DR of 300 – 400 MU/min should be respected for patients with PC. For patients with SCCscalp a lower DR (100 – 200 MU/min) should be chosen to minimize radiation dose given to serial OAR, particularly those who are located outside the end position of the leafs and inside the open field.

## Competing interests

The authors declare that they have no competing interests.

## Authors' contributions

DW participated in the design of the study and carried out the treatment plans. CFH participated in the draft of the manuscript. HV performed the statistical analysis. All authors read and approved the final manuscript.
